# Prophylactic Measures for Decay of the Teeth

**Published:** 1872-05

**Authors:** 


*Prophylactic Measures for Decay.*—Dr. J. Taft, at a recent meeting of the Ohio State Dental Society, in referring to Dr. Arthur's work, spoke as follows:

"The arrest of decay in its incipient stages, before filling is required, is, of course, very desirable. He starts out with the idea that the decay of the permanent teeth occurs from the lodgment of foreign substances upon them. His object is, to relieve this contact, or make space between them so as to keep them free from the lodgment of all foreign substances. He advocates the idea of beginning with the first permanent molars, so soon as they appear through the gums, and making a separation by cutting with a file or chisel, between it and the posterior temporary molar, by cutting off a portion of the temporary molar whether decayed or not, but more particularly if decayed. And when the central incisors come in, pursue the same course with them; so that the permanent teeth are kept free from the lodgment of any foreign substances, and so that cleanliness shall be an easy matter. This was a very important point and he had no doubt if this course was pursued it would reduce the amount of proximal decays to a small portion of what now exists. The attention of dentists in years gone by, had been given mostly ot disease after it has begun and gone on to a considerable extent. Dentists send patients away sometimes, refusing to fill cavities until they become larger and easier to fill. Such should not be the case. Members of the profession ought to study and investigate to ascertain what things deteriorate the teeth; what is liable to do so from the beginning, when the first rudiments are forming, and what may interrupt the perfect development of the teeth. This would involve histology, all the way from the beginning up. The formation of tissues, and everything connected with them, should be investigated, and such things employed as would control and modify them, so as to secure the best possible results. It is time people should awake to the importance of the growth and development of the teeth, and especially those who, to any extent, have anything to do with the subject of medicine and hygiene—all whose attention is directed in any way to the welfare of the body—should feel it their duty to educate the people in regard to these matters, and always and everywhere enter their protests against the vicious habits and customs of life so prevalent and tending so much to break down the race to a mere condition of dwarfage."

